# Syk inhibitors protect against microglia-mediated neuronal loss in culture

**DOI:** 10.3389/fnagi.2023.1120952

**Published:** 2023-03-15

**Authors:** Timothy J. Y. Birkle, Guy C. Brown

**Affiliations:** Department of Biochemistry, University of Cambridge, Cambridge, United Kingdom

**Keywords:** neuroinflammation, neurodegeneration, microglia, phagocytosis, Syk, neuroprotection, Alzheimer’s disease, Parkinson’s disease

## Abstract

Microglia are brain macrophages and play beneficial and/or detrimental roles in many brain pathologies because of their inflammatory and phagocytic activity. Microglial inflammation and phagocytosis are thought to be regulated by spleen tyrosine kinase (Syk), which is activated by multiple microglial receptors, including TREM2 (Triggering Receptor Expressed on Myeloid Cells 2), implicated in neurodegeneration. Here, we have tested whether Syk inhibitors can prevent microglia-dependent neurodegeneration induced by lipopolysaccharide (LPS) in primary neuron-glia cultures. We found that the Syk inhibitors BAY61-3606 and P505-15 (at 1 and 10 μM, respectively) completely prevented the neuronal loss induced by LPS, which was microglia-dependent. Syk inhibition also prevented the spontaneous loss of neurons from older neuron-glia cultures. In the absence of LPS, Syk inhibition depleted microglia from the cultures and induced some microglial death. However, in the presence of LPS, Syk inhibition had relatively little effect on microglial density (reduced by 0–30%) and opposing effects on the release of two pro-inflammatory cytokines (IL-6 decreased by about 45%, TNFα increased by 80%). Syk inhibition also had no effect on the morphological transition of microglia exposed to LPS. On the other hand, inhibition of Syk reduced microglial phagocytosis of beads, synapses and neurons. Thus, Syk inhibition in this model is most likely neuroprotective by reducing microglial phagocytosis, however, the reduced microglial density and IL-6 release may also contribute. This work adds to increasing evidence that Syk is a key regulator of the microglial contribution to neurodegenerative disease and suggests that Syk inhibitors may be used to prevent excessive microglial phagocytosis of synapses and neurons.

## Introduction

Microglia are resident macrophages of the central nervous system (CNS) and are increasingly implicated in brain pathologies such as Alzheimer’s disease (AD), Parkinson’s disease, stroke, brain trauma, schizophrenia, autism, retinal and spinal pathology, as well as brain ageing (Colonna and Butovsky, 2017; Qin et al., 2019; Greenhalgh et al., 2020; Kwon and Koh, 2020; Blaylock and Faria, 2021). These dynamic cells carry out disease-modifying functions, including: phagocytosis, degradation, and compaction of amyloid plaques (Fu et al., 2012; Condello et al., 2015; Yuan et al., 2016; Feng et al., 2020; Huang et al., 2021; Lemke and Huang, 2022); regulation of inflammatory states (Heneka et al., 2013); phagocytosis of synapses (Stevens et al., 2007; Schafer et al., 2012; Hong et al., 2016); phagocytosis of dead cells and debris (Sierra et al., 2010; Anderson et al., 2022); phagocytosis of stressed neurons (Fricker et al., 2012a,b); and recruiting peripheral immune cells to the brain (Zhang et al., 2022). Thus, it is important to better understand the intracellular signalling pathways controlling these contrasting functions of microglia.

Spleen tyrosine kinase (Syk), a cytosolic tyrosine kinase first cloned over 30 years ago (Taniguchi et al., 1991), is expressed by microglia (Satoh et al., 2012; Ennerfelt et al., 2022) and mediates inflammatory and/or phagocytic responses induced by cell surface receptors (Mócsai et al., 2010). Syk is activated by binding to phosphorylated ITAM (immunoreceptor tyrosine-based activation motif) domains of ligated cell surface receptors (Dectin-1, FcγRIIA) or adaptors (DAP12, FcR γ chain) of cell surface receptors (Fcγ receptors; TREM2; complement receptor 3, CR3; colony stimulating factor 1 receptor, CSF1R) (Mócsai et al., 2010; Walbaum et al., 2021). Some of these receptors are particularly relevant to disease: TREM2 is a phagocytic receptor mediating microglial phagocytosis of amyloid plaques (Yuan et al., 2016), synapses (Filipello et al., 2018), and neurons (Linnartz-Gerlach et al., 2019; Popescu et al., 2022), and TREM2 variants can confer risk for AD and other neurodegenerative diseases (Zhou et al., 2019). Similarly, CR3 mediates microglial phagocytosis of synapses during both development and neurodegeneration (Stevens et al., 2007; Schafer et al., 2012; Hong et al., 2016; Vasek et al., 2016). In contrast, CSF1R maintains microglial proliferation, and variants cause developmental CNS pathology (Gerber et al., 2018; Sosna et al., 2018; Mancuso et al., 2019; Hume et al., 2020; Cheng et al., 2021). Activated Syk then signals via PI3K/AKT, JNK, and phospholipase-C γ2 (PLCG2, variants of which associate with AD risk) to promote cell survival, proliferation and phagocytosis (Arndt et al., 2004; Sims et al., 2017; Tsai et al., 2020; Ennerfelt et al., 2022). Overall, Syk may be a hub of disease-relevant signalling in microglia.

Spleen tyrosine kinase has been shown to coordinate detrimental microglial activity in mouse models of tauopathy, brain trauma, stroke, and inflammation (Schweig et al., 2019; Ye et al., 2020; He et al., 2022; Kim et al., 2022). However, targetted deletion of the Syk gene in microglia exacerbated pathology in an amyloid model of AD and in a demyelinating model of multiple sclerosis, apparently by reducing microglial phagocytosis of amyloid and myelin debris (Ennerfelt et al., 2022). Syk expression is upregulated in amyloid models of AD (Sierksma et al., 2020), and Syk partly mediates induction of the disease-associated microglia (DAM) expression profile in such models (Ennerfelt et al., 2022). *SYK* gene variants are associated with AD risk, though not at genome-wide statistical significance (Sierksma et al., 2020). Thus, Syk is implicated in a variety of brain pathologies, but its mechanism of action and its overall beneficial/detrimental role in different brain pathologies is unclear.

Brain inflammation is associated with most brain pathologies, and there is evidence that chronic brain inflammation can be detrimental (DiSabato et al., 2016). Microglia are key mediators of this brain inflammation, and microglia activated by inflammation can damage neurons (Kwon and Koh, 2020). Inflammation greatly increases microglial phagocytosis, and one mechanism by which activated microglia are damaging is via excessive microglial phagocytosis of live synapses and neurons (Fricker et al., 2012b; Hong et al., 2016; Golia et al., 2019). Thus, it is important to find treatments that block excessive microglial phagocytosis of live synapses and neurons. Syk mediates phagocytosis induced by a variety of microglial receptors, and Syk inhibitors block such phagocytosis (Crowley et al., 1997; Song et al., 2004; Gevrey et al., 2005; Scheib et al., 2012; Murakami et al., 2014; Yao et al., 2019; McQuade et al., 2020; Walbaum et al., 2021). Thus, it is of interest to know whether Syk inhibitors are neuroprotective via inhibition of microglial phagocytosis. There are safe and effective drugs to inhibit Syk in humans and mice, so if Syk inhibitors were protective, they could potentially be used as treatment.

Inflammation can be induced in body and brain by lipopolysaccharide (LPS) via activation of Toll-like receptor 4 (TLR4), and LPS levels are elevated in blood and brain in AD and other neurodegenerative conditions (Zhang et al., 2009; Zhan et al., 2016; Zhao et al., 2017). Thus, LPS is used to model brain inflammation, and this recapitulates transcriptomic signatures of microglia in AD (Monzón-Sandoval et al., 2022). In culture and *in vivo*, the neuronal loss induced by LPS can be prevented by blocking microglial phagocytosis (Fricker et al., 2012a). Recently, it was reported that LPS-induced neuronal loss in the brain can be prevented by a Syk inhibitor, and this was attributed to blockade of the inflammatory activation of microglia (Kim et al., 2022). However, the role of phagocytosis was not tested as this is challenging to do *in vivo*. Here, we investigated whether LPS-induced neuronal loss in culture could be prevented by Syk inhibitors, and if so by what mechanism. We tested this in primary neuron-glia cultures, in which LPS causes neuronal loss mediated by microglial inflammation and phagocytosis (Fricker et al., 2012b). We observed complete protection of neurons from neurodegeneration by two structurally unrelated Syk inhibitors, BAY61-3606 (at 1 μM) and P505-15 (at 10 μM). Our data indicate that Syk inhibition prevents excessive microglial phagocytosis of synapses and neurons, and this may explain the neuroprotection by Syk inhibition.

## Materials and methods

### Materials and general tissue culture

The following media and solutions were obtained from ThermoFisher: DMEM (41965062), PBS (70011051), HBSS (14175095), trypsin-EDTA (15400054), Versene (15040066). Penicillin/Streptomycin was from Sigma-Aldrich (P4333), as was poly-L-lysine (PLL; P4707), cytochalasin D (C8273), and LPS (L6143). Gentamicin (G38000) was from Melford. Multi-well F-bottom cell culture plates and T75 flasks were from Greiner Bio-One. Cell strainers were from Falcon (352340/352360). All cultures were maintained at 37°C in humidified 5% CO_2_ incubators and all media were supplemented with either penicillin (100 U/ml) and streptomycin (100 μg/ml), or gentamicin (50 μg/ml). All experiments using animal tissue complied with the UK Animals (Scientific Procedures) Act 1986 and were approved by the local ethical committee at Cambridge University.

### Syk inhibitors

BAY61-3606 was obtained from Cayman Chemical (CAY11423), dissolved in DMSO, and aliquoted into single-use aliquots prior to use. P505-15 (also known as PRT062607) was from MedChem Express (HY-15323) in DMSO-dissolved format and was aliquoted into single-use aliquots prior to use.

### Primary cerebellar neuron-glia cultures

Neuron-glia cultures were prepared as previously described (Kinsner et al., 2005). Briefly, P3-6 rat cerebella were dissected in ice-cold HBSS, finely diced, and incubated in Versene for 5–10 min at 37°C. Cells were then mechanically dissociated by trituration, transferred to warm media, pelleted, resuspended, and 40 μm-strained. Live cells were counted using trypan blue and cultures were seeded at 295,000 live cells/cm^2^ in multi-well plates coated with 0.01% PLL. After 24 h, debris was shaken off manually and the media replaced. Mixed cultures were treated after at least 7 days *in vitro* (*DIV*). Coculture media consisted of high-glucose DMEM supplemented with 5% certified foetal bovine serum (FBS; Gibco; 10082147), 5% horse serum (Gibco; 26050088), 2 mM L-glutamine, 13 mM glucose, 5 mM HEPES, and 20 mM KCl.

### Neuronal loss assays

Neuron-glia cultures were seeded at 100,000 cells/well in 0.01% PLL-coated 96-well plates. To assay for effects on inflammatory neuronal loss, cultures were treated at *DIV7* with Syk inhibitors at the stated concentrations for 30 min prior to the addition of LPS at 100 ng/ml. For corresponding microglial depletion experiments, *DIV6* neuron-glia cultures were treated with 25 mM L-leucine methyl ester (LME; L1002) for 1 h as previously published (Jebelli et al., 2015), omitting washes before the final media replacement. Further treatments were carried out as usual from *DIV7-10*. To assay for effects on spontaneous neuronal loss with age, cultures were treated with Syk inhibitors from *DIV9-12* instead.

Some culture media was removed at the end of treatment (and saved for analysis where necessary), prior to staining of cultures through topical addition of staining stock into a final volume of 50 μl. The neuron-glia cultures were stained for 1 h with (final concentrations): NeuroFluor NeuO (Er et al., 2015; 200 nM; 01801), Isolectin IB_4_-AF594 (2 μg/ml; I21413) and Hoechst 33342 (1 μg/ml; 62249). Without removal of staining media, cultures were then imaged using the 10× objective on an EVOS M5000 epifluorescence microscope, with four images taken in consistent positions around each well. Image sets were analysed for the number of each cell type present using a custom CellProfiler (Stirling et al., 2021b)/CellProfiler Analyst (Stirling et al., 2021a) pipeline for which in-house validation demonstrates 90–95% accurate classification of each cell type in the neuron-glia cultures.

### Syk immunocytochemistry

Neuron-glia cultures in 96-well plates were fixed in 4% paraformaldehyde (28906) for 10 min at room temperature, then washed once with 50 mM NH_4_Cl-containing PBS and twice with standard PBS. Cells were then permeabilised for 10 min with 0.1% TX-100 in PBS, washed three times, blocked with 5% goat serum in PBS for 1.5 h, and stained overnight at 4°C with 1:200 mouse α-Syk (626202) and 1:200 rabbit α-Iba1 (019-19741) primary antibodies. Cells were then washed three times, followed by 1 h room temperature incubation with 1 μg/ml Hoechst 33342, and 1:1,000 goat α-mouse AF568 (A11004) and 1:1,000 goat α-rabbit AF488 (A11008) secondary antibodies. After three more washes, cells were imaged using the 20× objective on an EVOS M5000 microscope.

### Analysis of ghost microglia

Triculture images were pre-processed with rolling ball background subtraction on all channels using a custom ImageJ (Schneider et al., 2012) macro. IB_4_-positive objects were then identified using cell detection on QuPath (Bankhead et al., 2017), and objects lacking DNA were then identified by thresholding based on the total Hoechst fluorescence signal within each object.

### Microglial morphology analysis

Images pre-processed with ImageJ were imported into QuPath for nuclei segmentation and microglial classification. Through a cross-platform script between QuPath and ImageJ, marker-based watershed from the MorphoLibJ plugin library (Legland et al., 2016) was used to flood-fill auto-thresholded microglial cell body masks from microglial nuclei, and each resulting microglial object (typically around 2,000 per technical replicate per condition) was analysed using ImageJ shape measurements.

### Condensed nuclei within microglia analysis

Images pre-processed with ImageJ were imported into QuPath for nuclei segmentation and classification of condensed nuclei based on having small size and high Hoechst staining intensity. Nuclei within microglia were further determined based on IB_4_-AF594 staining intensity in and around the nucleus area.

### Primary glial cultures

Microglia-astrocyte glial cultures, from which pure microglial cultures were obtained, were prepared as previously described (Bal-Price and Brown, 2001). Briefly, P3-6 rat cerebral cortices were dissected, diced, and incubated at 37°C in 0.033% trypsin (diluted in HBSS) for 15 min. Cells were then mechanically dissociated by trituration, 100 μm- and 40μm-strained, and finally seeded at a ratio of 1 brain (2 cortices) worth of cells per T75 flask coated with 0.005% PLL. After 24 h, debris was shaken off and media replaced. From *DIV7* and 18–24 h prior to experiments, microglia were shaken off, resuspended in 1-part conditioned media: 2-parts fresh, then seeded as needed in 0.005% PLL-coated plates. Glial media was DMEM supplemented with 10% certified FBS.

### Primary microglia cell death assay

Primary microglia were seeded at 50,000 cells/well in 0.005% PLL-coated 96-well plates and allowed to settle over 4–6 h. Cells were then treated overnight for 18–24 h prior to 1 h staining with Hoechst 33342 (1 μg/ml), Isolectin IB_4_-AF488 (4 μg/ml; I21411), and propidium iodide (PI; 2 μg/ml; P4170). After microscopy (EVOS M5000), images were pre-processed (background-subtracted and smoothed) using a custom ImageJ macro and nuclei were segmented and classified for cell death-relevant staining using QuPath.

### Primary microglia phagocytosis assays

Primary microglia were seeded at 25,000 cells/well in PLL-coated 48-well plates and allowed to settle of 4–6 h. Cells were then treated overnight with Syk inhibitors and LPS at the specific concentrations. Cytochalasin D treatments involved addition to 5 μM, 2 h prior to target addition. Targets used were 5 μm fluorescent beads (Spherotech; CFP-5070-2; 10 μl of 0.025% w/v beads per well) and pHrodo-labelled synaptosomes (16 μg/well). In all cases, phagocytic targets were dispersed throughout wells via both pipetting and gentle plate agitation. After target incubation for 2 h, media was aspirated, microglia resuspended in ice-cold PBS, and cells were transferred to microfuge tubes and kept on ice for subsequent flow cytometry analysis.

All flow cytometry was carried out on a BD CytoFlex S instrument. DAPI was spiked into each sample at 1 μg/ml final concentration 1 min prior to analysis, and standard gating was used: cells gated by FSC-A/SSC-A; singlets gated by FSC-A/FSC-H; live cells gated by lack of DAPI staining. In synaptosome experiments, phagocytic cells were gated using a gate with a 1% positive rate for the cytochalasin D negative controls. In 5 μm bead experiments, any bead uptake caused a large shift in fluorescence and gate boundaries were therefore set halfway between the clearly distinct populations. At least 5,000 events were recorded for each sample.

### Synaptosome preparation

Synaptosomes were prepared from juvenile rat cortices: tissue was homogenised by Dounce homogenisation and synaptosomes purified using Percoll gradient centrifugation according to a published protocol (Dunkley et al., 2008), then frozen in liquid nitrogen. Protein concentrations were measured via Nanodrop. For experiments, synaptosomes were thawed and transferred to H-KRP buffer, pelleted at 20,000 *g* for 5 min, and resuspended for staining with pHrodo (10 μM in H-KRP buffer; Invitrogen; P35372) for 15 min at 37°C. Synaptosomes were then washed and resuspended in buffer. H-KRP buffer was an aqueous solution of: 143 mM NaCl, 4.7 mM KCl, 1.3 mM MgSO_4_, 1.2 mM CaCl_2_, 20 mM HEPES (stock at pH 7.4 with saturated Tris), 0.1 mM Na_2_HPO_4_, and 10 mM D-glucose. This was prepared fresh, 0.22 μm-filtered, and infused with CO_2_ in a 5% CO_2_ incubator for 60 min prior to use.

### BV2 cell culture

BV2 cells were cultured in DMEM with 10% FBS (Sigma-Aldrich; F9665) in T75 flasks, passaging using 0.05% trypsin-EDTA.

### Western blotting

For validation of Syk inhibition, BV2 cells were resuspended in un-supplemented DMEM and seeded at 500,000 cell/well in 6-well plates. Twenty-four hours later, wells were pre-treated with Syk inhibitors at the stated concentrations for 2 h prior to 10 min treatment with 50 μg/ml Concanavalin A (ConA; C5275). For measurement of Homer1 protein levels in neuron-glia cultures, cells were plated in six-well plates and treatments performed as described.

In either case, media was then quickly aspirated and 100 μl of lysis buffer added to each well. Lysis buffer consisted of 1% TX-100, 150 mM NaCl, 50 mM Tris (pH 8), supplemented with protease and phosphatase inhibitors (11697498001; 4906845001) at recommended concentrations. Lysates were collected with scraping, lysed on ice for 20 min with intermittent vortexing, and then cleared at 15,000 *g* for 15 min at 4°C prior to storage at −20°C. About 19.5 μl of lysates were mixed with 3 μl 10× reducing agent (NP0004) and 7.5 μl NuPAGE loading buffer (NP0007), denatured at 90°C for 10 min, and then loaded onto a 4–12% Bis-Tris NuPAGE precast gel (NP0321) alongside a fluorescent protein ladder (928-40000). After iBlot transfer onto PVDF, blots were blocked in 5% milk TBST for 1 h before overnight staining at 4°C with primary antibodies in 5% milk TBST. After 3× washes with TBST (15, 5, and 5 min), blots were stained with secondary antibodies, then washed again before imaging and analysis using a LI-COR Odyssey CLx instrument.

For pSyk BV2 blots, primary antibodies were 1:1,000 diluted rabbit α-pSyk (Tyr525/526; MA5-14918) and 1:5,000 diluted mouse α-β-actin (66009-1) antibodies, and secondary antibodies were 1:5,000 diluted α-rabbit IRDye 800CW (925-32213) and 1:20,000 diluted α-mouse AF680 (A10038). For Homer1 neuron-glia blots, rabbit α-Homer1 (160003) and 1:1,000 mouse α-NeuN (MAB377) primary antibodies were diluted 1:1,000, and α-rabbit IRDye 800CW and α-mouse AF680 secondary antibodies were diluted 1:1,0000.

### Supernatant cytokine ELISA assays

Supernatants were collected from each well of 96-well plate cultures (treated in technical triplicates) and each replicate supernatant was tested once. Standards were each assayed in triplicate. All standard curves were fitted and interpolated using 4PL non-linear regression on GraphPad Prism and achieved *R*^2^ > 0.99. ELISA assays provided without pre-coated plates were carried out in Maxisorp plates (ThermoFisher; 442404), and all absorbance measurements were made using a FlexStation 3 instrument (Molecular Devices). IL-6 measurement: a BioLegend LEGEND MAX™ Rat IL-6 ELISA Kit (437107) was used according to the provided protocol, with 5 μl of supernatant tested per well. TNFα measurement: a BioLegend ELISA MAX™ Deluxe Set Rat TNF-a Kit (438204) was used according to the provided protocol, with 10 μl of supernatant tested per well. IL-1β measurement: an Invitrogen IL-1β Rat Uncoated ELISA Kit (88-6010-22) was used according to the provided protocol, with 40 μl of supernatant tested per well.

### Software and statistics

All custom macros for ImageJ and QuPath, pipelines for CellProfiler, and CellProfiler Analyst classifiers can be found on Github under the user *timjyb.* CellProfiler 4.2.4 (Stirling et al., 2021b) was built from source and the neuron-glia cultures analysis pipeline included a Cellpose 2.1.0 module (Stringer et al., 2021). CellProfiler Analyst 3.0 (Stirling et al., 2021a) was used for trained cell classification. For other image analysis, ImageJ 1.52-1.53 (Schneider et al., 2012; Schindelin et al., 2015) via FIJI (Schindelin et al., 2012) and QuPath 0.3.0 (Bankhead et al., 2017) were used.

Statistical analyses were performed using GraphPad Prism 9 for Windows (GraphPad Software, San Diego, CA, USA). For each experimental condition, at least three technical replicates were performed, using the mean for analysis. At least three biological repeats were performed per experiment: for primary cell experiments, these were distinct preparations of cells from different rat litters; for BV-2 cell line experiments, these represent distinct passages and/or flasks of cells. Most data were analysed as repeat-wise matched data by one- or two-way ANOVA (or equivalent mixed-effects models), while assuming circularity/sphericity. Matching was advisable given the repeat-wise variability that is to be expected in primary cell preparations; this preserves power where there is such variation, and retains power in the absence of it as well (Lew, 2007). Where only two conditions were compared, paired t-tests were used instead. *Post-hoc* tests were as stated in figure legends.

## Results

### Syk inhibitors prevent LPS-induced neuronal loss

**FIGURE 1:**
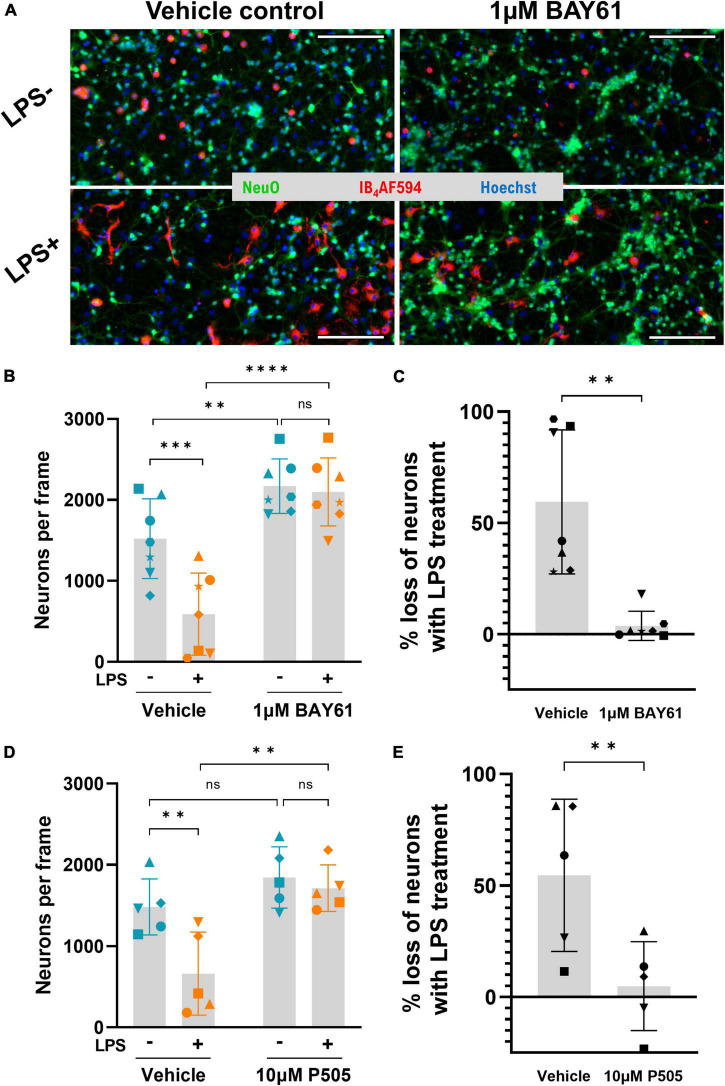
Syk inhibitors prevent LPS-induced neuronal loss in primary neuron-glia cultures. **(A)** Representative 10× images (cropped) of primary rat cerebellum neuron-glia cultures treated ± BAY61 (1 μM) ± LPS (100 ng/ml) for 3 days and stained with Hoechst 33342 (nuclei), IB_4_-AF594 (microglia), and NeuO (live neurons). Scale bars = 100 μm. **(B)** Average neuronal counts per image in cultures treated ± BAY61 (1 μM) and ± LPS (100 ng/ml) for 3 days (DIV10). RM 2-way ANOVA with Šídák’s *post-hoc* test. **(C)** LPS-induced neuronal loss ± BAY61 calculated from the neuronal counts of **(B)** as percentage of neuronal counts in absence of LPS. Paired *t*-test. **(D)** Average neuronal counts per image in cultures treated ± P505 (10 μM) and ± LPS (100 ng/ml) for 3 days (DIV10). RM 2-way ANOVA with Šídák’s *post-hoc* test. **(E)** LPS-induced neuronal loss ± P505 from the neuronal counts of **(D)** as percentage of neuronal counts in absence of LPS. Paired *t*-test. All panels: each datapoint represents the mean of three technical replicates. Number of biological repeats = number of datapoints per column. **p < 0.01, ***p < 0.001, ****p < 0.0001.

We first tested whether Syk inhibitors could protect against LPS induced neuronal loss in neuron-glia cultures from rat cerebellum, treated ± 100 ng/ml LPS ± Syk inhibitors over 3 days. Each cell type was counted after staining with Hoechst 33342 (DNA), NeuO (Er et al., 2015; live neurons), and AF594-conjugated Isolectin GS-IB_4_ (IB_4_-AF594; microglia). LPS induced significant neuronal loss, as previously reported (Fricker et al., 2012b; Figures 1A, B). BAY61-3606 (referred to as BAY61 hereafter) is a specific Syk inhibitor (Yamamoto et al., 2003; see Supplementary Figure 1A for structure) and, strikingly, cotreatment with 1 μM BAY61 completely prevented this neuronal loss (Figures 1A–C). The same changes were observed when quantifying total cell count, confirming that the changes in neuronal number are genuine, rather than reflecting changes in neuronal differentiation or NeuO staining efficacy because of LPS and/or BAY61 treatment (Supplementary Figure 2).

BAY61 treatment also increased neuronal density in the absence of LPS (Figures 1A, B). This suggests that there is some spontaneous neuronal loss in these cultures, which were treated from 7 to 10 days after isolation from neonatal rat brain, and that BAY61 prevented this loss. To test this, live neurons were counted in neuron-glia cultures 7, 9/10, and 12 days after isolation. This confirmed that there was a spontaneous loss of neurons, which was more marked in the older cultures (Supplementary Figures 3A–D). Treatment of these older cultures with BAY61 from day 9 to day 12 after isolation reduced the spontaneous neuronal loss (Supplementary Figure 2E). Thus, BAY61 protects against both spontaneous and LPS-induced neuronal loss.

To limit the possibility that protection by BAY61 was due to off-target effects, we also tested an alternative and more specific Syk inhibitor, P505-15 (Coffey et al., 2012; hereafter, P505; see Supplementary Figure 1B for structure). P505 also completely prevented neuronal loss, albeit at a higher concentration of 10μM (Figures 1D, E).

To confirm that Syk was indeed inhibited by BAY61 at 1 μM and P505 at 10 μM, we measured Syk autophosphorylation on its Tyr519/520 residues (equivalent to human Syk Tyr525/526 residues) using Western blot after receptor stimulation of BV2 microglia with Concanavalin A. This confirmed that both inhibitors reduced Syk autophosphorylation at the concentrations used (Supplementary Figure 4).

### Both Syk and microglia are necessary for LPS-induced neuronal loss

**FIGURE 2:**
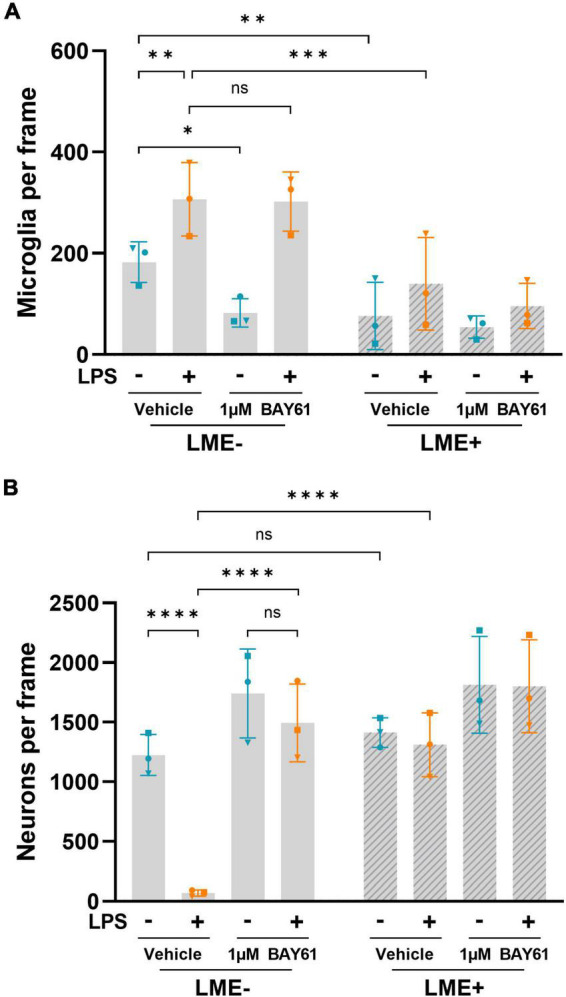
LPS-induced neuronal loss is dependent on microglia. **(A)** Average microglial counts per image in neuron-glia cultures treated ± BAY61 (1 μM) and ± LPS (100 ng/ml) for 3 days (DIV10), after previous ± LME treatment to deplete microglia. **(B)** Average DIV10 neuronal counts per image in neuron-glia cultures treated ± BAY61 (1 μM) and ± LPS (100 ng/ml) for 3 days (DIV10), after previous ± LME treatment to deplete microglia. All panels: each datapoint represents the mean of 3 technical replicates. Number of biological repeats = number of datapoints per column. *p < 0.05, **p < 0.01, ***p < 0.001, ****p < 0.0001. RM 2-way ANOVA with Šídák’s *post-hoc* test.

To test which cells in the neuronal-glial cultures expressed Syk, we stained the cultures using antibodies against Syk. Syk was expressed by both microglia and neurons in these cultures, with stronger expression in microglia (Supplementary Figure 5). To distinguish the effects of microglial Syk in these cultures, we depleted cultures of microglia using L-leucine-methyl-ester (LME; Jebelli et al., 2015) prior to LPS treatment. We confirmed that LME depleted microglia (Figure 2A) with a small effect on astrocyte density (Supplementary Figure 6A), and no effect on neuronal density (Figure 2B). Importantly, this microglial depletion completely prevented LPS-induced neuronal loss (Figure 2B). Note that the LPS-induced neuronal loss, in the absence of Syk inhibition, was stronger in these experiments (Figure 2B) that the previous experiments (Figure 1), possibly due to cultures being stressed by the media swaps involved in LME treatment. However, even in these conditions where LPS caused almost complete neuronal loss, 1μM BAY61 entirely prevented this (Figure 2B). This shows that inflammatory neuronal loss in these neuron-glia cultures requires microglia and Syk activity, while suggesting that Syk inhibition could be neuroprotective itself by depleting microglia.

### Syk inhibitors partially deplete microglia by inducing microglial cell death

**FIGURE 3:**
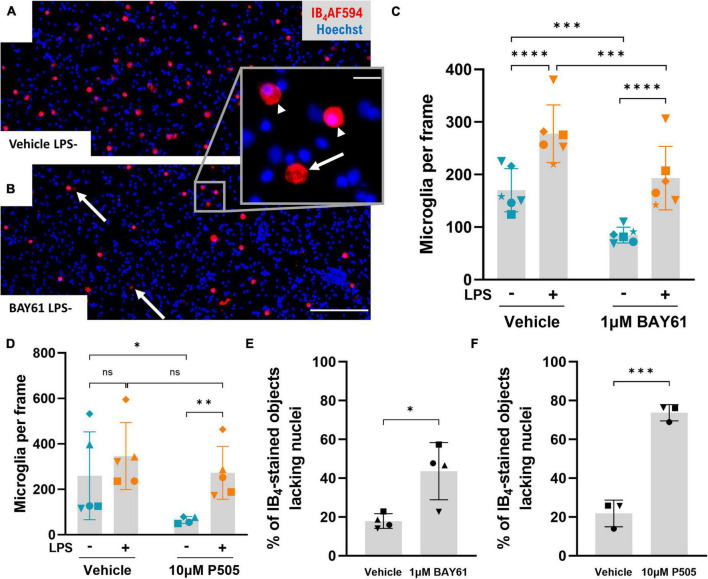
Syk inhibitors deplete microglia in the absence of LPS. **(A,B)** Representative 10x images (cropped) of LPS-untreated primary rat neuron-glia cultures treated ± BAY61 (1 μM) for 3 days and stained with Hoechst 33342 and IB_4_-AF594. Scale bar = 100 μm. Arrows indicate ghost-like microglia (IB_4_-stained objects lacking any nuclear staining). **(B)** Magnified view of a ghost-like microglia (arrow) adjacent to intact microglia (indicated by arrowheads). Scale bar = 10 μm. **(C)** Average microglial counts per image in neuron-glia cultures treated ± BAY61 (1 μM) and ± LPS (100 ng/ml) for 3 days (DIV10). RM 2-way ANOVA with Šídák’s *post-hoc* test. **(D)** Average microglial counts per image in neuron-glia cultures treated ± P505 (10 μM) and ± LPS (100 ng/ml) for 3 days (DIV10). RM mixed-effects analysis with Šídák’s *post-hoc* test. **(E)** Percentage of IB_4_-stained objects which lack nuclear DNA staining in images from LPS-untreated neuron-glia cultures treated ± BAY61 (1 μM) for 3 days (DIV10). Paired *t*-test. **(F)** Percentage of IB_4_-stained objects which lack nuclear DNA staining in images from LPS-untreated neuron-glia cultures treated ± P505 (10 μM) for 3 days (DIV10). Paired *t*-test. All panels: each datapoint represents the mean of three technical replicates. Number of biological repeats = number of datapoints per column. *p < 0.05, **p < 0.01, ***p < 0.001, ****p < 0.0001.

To investigate whether Syk inhibition depletes microglia in the neuron-glia cultures, we analysed microglial density in the original experiment of Figure 1. In the absence of LPS, both BAY61 and P505 significantly reduced microglial numbers in the neuron-glia cultures (Figures 3A–D). However, P505 failed to deplete microglia from LPS-treated neuron-glia cultures, and for BAY61 the depletion with LPS present was only modest, though significant (Figures 3A–D). Additionally, in the experiments of Figure 2 BAY61 did not reduce microglial density in the presence of LPS at all (Figure 2A). Neither LPS nor P505 affected the number of astrocytes in the neuron-glia cultures; however, BAY61 produced a small but significant decrease for astrocytes (Supplementary Figures 7A, B).

Further examination of the neuron-glia cultures revealed IB_4_-stained cellular structures lacking nuclei (Figure 3B, inset). The focal plane’s depth of field was about 10 μm, larger than the width of a microglia, and adjustment of the focal plane did not reveal out-of-plane nuclei (not shown). The lectin IB_4_ binds to microglial cell membranes and therefore these “ghost-like” microglia may represent microglia that have necrosed, releasing nucleus and cytoplasm but leaving cell membrane adhered to the culture plate. This is reminiscent of the ‘ghost tangles’ of tau that remain after neuronal death in Alzheimer’s disease (DeTure and Dickson, 2019). In neuron-glia cultures not treated with LPS, the prevalence of ghost-like microglia more than doubled after either BAY61 or P505 treatment, suggesting that these treatments induce microglial death (Figures 3A, B, E, F).

**FIGURE 4:**
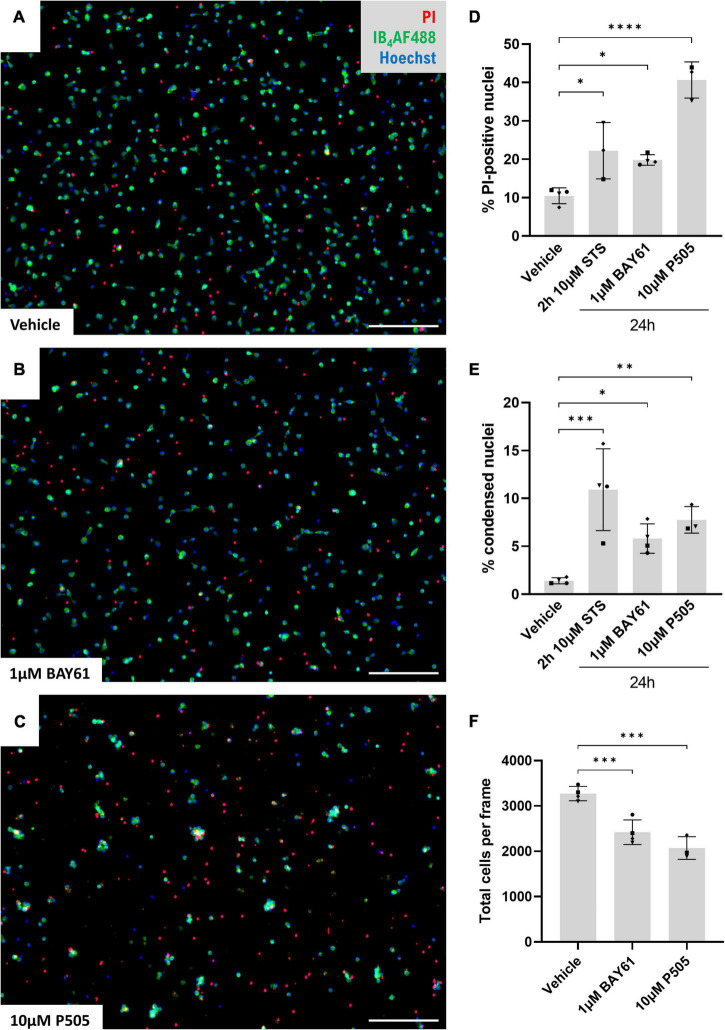
Microglial cell death is induced by Syk inhibitors. **(A–C)** Representative 10× images (cropped) of primary rat cortical microglia treated with ± BAY61 (1 μM) or ± P505 (10 μM) for 24 h and stained with Hoechst, IB_4_-AF488, and propidium iodide (PI). Scale bars = 100 μm. **(D)** Average percentage PI-positive nuclei in images of primary rat cortical microglia treated with BAY61 (1 μM) or P505 (10 μM) for 24 h, or staurosporine (10 μM) for 2 h. **(E)** Average percentage condensed nuclei in images of primary rat cortical microglia treated with BAY61 (1 μM) or P505 (10 μM) for 24 h, or staurosporine (10 μM) for 2 h. **(F)** Average total cells in images of primary rat cortical microglia treated with BAY61 (1 μM) or P505 (10 μM) for 24 h. All panels: each datapoint represents the mean of three technical replicates. Number of biological repeats = number of datapoints per column. *p < 0.05, **p < 0.01, ***p < 0.001, ****p < 0.0001. RM mixed-effects analysis with Dunnett’s *post-hoc* test.

To test whether Syk inhibition causes microglial cell death independent of other cell types, we next used cultures of isolated cortical microglia treated over 24 h and imaged after staining with Hoechst 33342 (to stain all nuclei) and propidium iodide (PI, for nuclei of necrotic cells). 1 μM BAY61 and 10 μM P505 caused significant increases in the percentage of PI-positive cells, comparable to or greater than the increase in cultures treated with staurosporine (STS; an inducer of apoptosis; Figures 4A–D). We also observed increases in the number of cells negative for PI staining, but with a condensed and apoptotic nuclear morphology (Figure 4E), indicating that Syk inhibitors induce some microglial apoptosis. There was also a decrease in the total number of microglial cells (Figure 4F). Overall, Syk inhibition clearly reduces microglial survival in the absence of LPS.

However, in the neuron-glia cultures treated with LPS, the Syk inhibitors caused either 0% (Figure 2A), 20% (Figure 3D) or 30% microglial depletion (Figure 3C), so microglial depletion cannot explain the full neuroprotection by the Syk inhibitors in these conditions (Figures 1B, D, 2B). Thus, we looked for other effects of the Syk inhibitors on microglia in neuron-glia cultures that might explain the neuroprotection.

### Syk inhibition does not change microglial morphology

**FIGURE 5:**
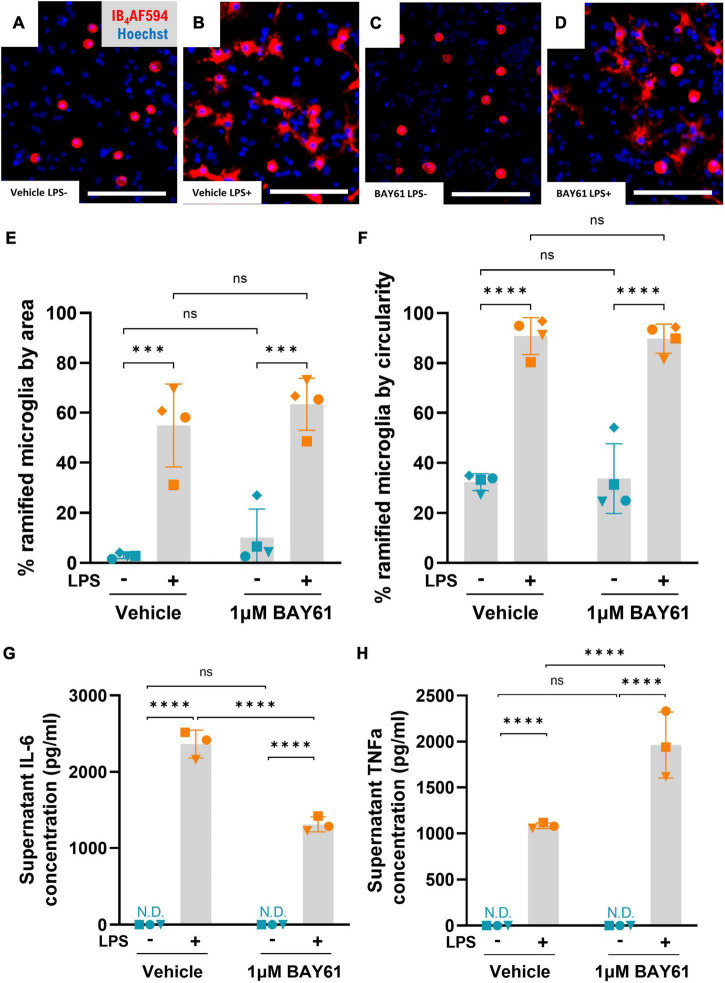
Syk inhibition has a limited effect on microglial inflammation. **(A–D)** Representative 10× images (cropped) of primary rat neuron-glia cultures treated ± BAY61 (1 μM) and ± LPS (100 ng/ml) for 3 days and stained with Hoechst 33342 and IB_4_-AF594. Scale bars = 50 μm. **(E)** Average percentage of microglia that are ramified in images of neuron-glia cultures treated with ± BAY61 (1 μM) and ± LPS (100 ng/ml) for 3 days (DIV10), as defined by cell area. **(F)** Average percentage of microglia that are ramified in images of neuron-glia cultures treated with ± BAY61 (1 μM) and ± LPS (100 ng/ml) for 3 days (DIV10), as defined by cell circularity. **(G)** Average concentration of IL-6 in supernatants from neuron-glia cultures treated ± BAY61 (1 μM) and ± LPS (100 ng/ml) for 3 days (DIV10). **(H)** Average concentration of TNFα in supernatants from neuron-glia cultures treated ± BAY61 (1 μM) and ± LPS (100 ng/ml) for 3 days (DIV10). All panels: each datapoint represents the mean of three technical replicates. Number of biological repeats = number of datapoints per column. ***p < 0.001, ****p < 0.0001. N.D. = not detectable. RM 2-way ANOVA with Šídák’s *post-hoc* test.

We next assessed the effect of Syk inhibition on general inflammatory phenotypes after LPS treatment of neuron-glia cultures, first quantifying microglial morphology. Cultured microglia treated with LPS attach to the cell culture well, then flatten down and ramify across the surface (Figures 5A, B), as previously described (Wollmer et al., 2001; Lively and Schlichter, 2018). We quantified this morphological transition by annotating all microglia (>10,000 per repeat) and setting thresholds to define: (i) an increase in cell area and (ii) a decrease in cell circularity (due to extension of processes). LPS strongly increased microglial ramification by either measure, as expected, but microglial morphology was unchanged by Syk inhibition with BAY61 either with or without LPS (Figures 5A–F). Thus, Syk inhibition has no effect on the morphological activation induced by LPS.

### Syk inhibition alters pro-inflammatory cytokine release

Cytokine release is another hallmark of inflammation, and we confirmed that LPS greatly increased release of IL-6 and TNFα (Figures 5G, H). However, there was no detectable release of IL-1β (data not shown). Cytokine levels in the absence of LPS were undetectable ± BAY61 treatment. However, in the presence of LPS, BAY61 significantly reduced IL-6 levels while significantly increasing TNFα release (Figures 5G, H. We note that BAY61 reduced microglial density in these cultures by about 30% (Figure 3C), so the decrease in IL-6 (by about 45%) may partially reflect fewer microglia being present, rather than a reduction in IL-6 release per cell. The IL-6 level per microglia (at the end of the culture) was reduced by about 20% by BAY61, and the TNFα level per microglia was increased by about 150%. Thus, BAY61 mildly reduced LPS-induced IL-6 release per microglia, and increased the release of proinflammatory TNFα per microglia. Nonetheless, the reduction in IL-6 levels is significant and could contribute to the neuroprotection given that IL-6 has been implicated in neuronal loss previously (Conroy et al., 2004).

### Syk inhibition reduces microglial phagocytosis

**FIGURE 6:**
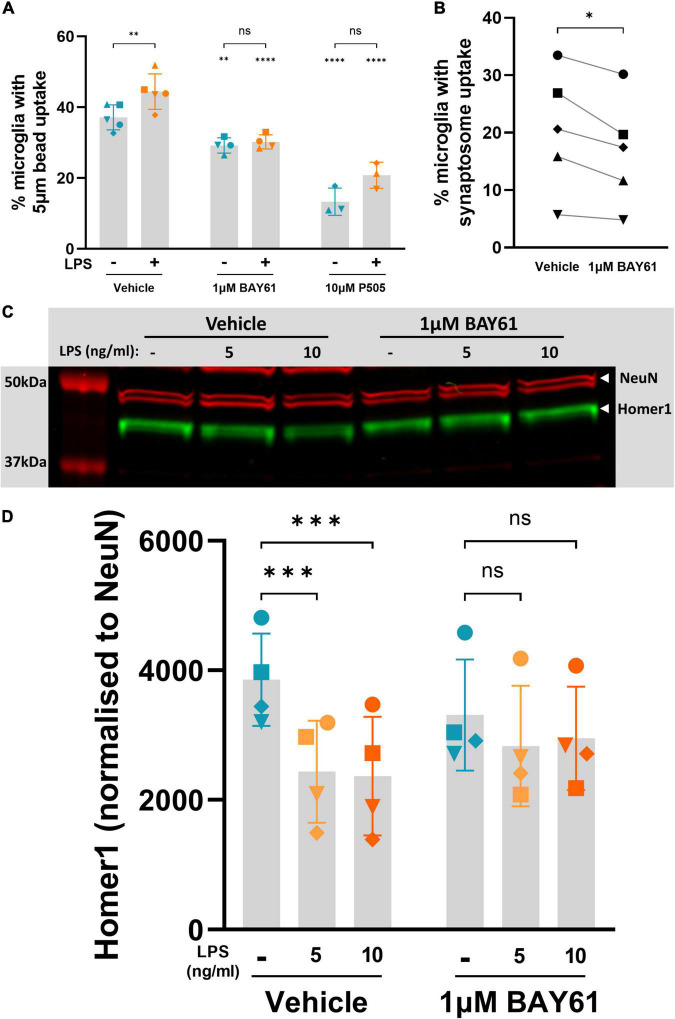
Syk inhibition decreases microglial phagocytosis of synaptic material. **(A)** Average percentage of non-necrotic microglia that took up fluorescent 5 μm beads over 2 h, after 24-h treatment ± BAY61 (1 μM) or ± P505 (10 μM), and ± LPS (100 ng/ml), as measured by flow cytometry. RM mixed-effects analysis with Šídák’s *post-hoc* test. **(B)** Average percentage of non-necrotic microglia that took up pHrodo-labelled synaptosomes over 2 h, after 24-h treatment ± BAY61 (1 μM), as measured by flow cytometry. Paired *t*-test. **(C)** Representative Western blot on lysates from neuron-glia cultures treated ± BAY61 (1 μM) and ± LPS (5-10 ng/ml) for 3 days (DIV10). Red = NeuN, green = Homer1. **(D)** Homer1 signal normalised against NeuN signal from Western Blots of lysates from neuron-glia cultures treated ± BAY61 (1 μM) and ± LPS (5–10 ng/ml) for 3 days (DIV10). RM 2-way ANOVA with Šídák’s *post-hoc* test. Datapoints in panels **(A,B)** represent the mean of three technical replicates. All panels: Number of biological repeats = number of datapoints per column. *p < 0.05, **p < 0.01, ***p < 0.001, ****p < 0.0001.

Previous work has shown that microglial phagocytosis of neurons is a primary cause of LPS-induced neuronal loss in these neuron-glia cultures (Neher et al., 2011; Fricker et al., 2012b). We therefore tested whether Syk inhibition blocks LPS-induced microglial phagocytosis by quantifying microglial phagocytosis of beads over 2 h. Both BAY61 and P505 reduced this phagocytosis in the presence and absence of LPS, and reduced the LPS-induced increase in phagocytosis (Figure 6A). We then tested microglial phagocytosis of a more physiological target: synaptosomes prepared from mouse brains, which are essentially isolated synapses (Dunkley et al., 2008). We note the large variability in synaptosome uptake between experimental repeats; this may be due to variation in synaptosome preparation and staining, which was performed independently for each experiment. Regardless, BAY61 reduced synaptosome uptake by cortical microglia within each experiment, and this reduction was significant (Figure 6B). Thus, Syk inhibitors reduce phagocytosis by isolated microglia of beads and isolated synapses.

As Syk inhibitors reduced phagocytosis of isolated synapses by isolated microglia, we tested whether Syk inhibition could reduce loss of neuronal synapses in the neuronal-glial cultures. In these cultures, low dose LPS (5 or 10 ng/ml) can induce synaptic loss with only mild neuronal loss (Supplementary Figure 8A). Synaptic density in the cultures was estimated 3 days after LPS treatment ± BAY61 by measuring Homer1 levels (a post-synaptic marker) by Western blot and normalising to NeuN levels (as a measure of neuronal density specifically, rather than total protein). Bands for Homer 1 and NeuN could be identified in the blots at the expected molecular weights (Figure 6C) despite some non-specific bands (Supplementary Figure 8B). Both 5 and 10 ng/ml LPS induced significant loss of normalised Homer1 levels, and this was reduced by BAY61 such that the loss was no longer significant (Figure 6D).

**FIGURE 7:**
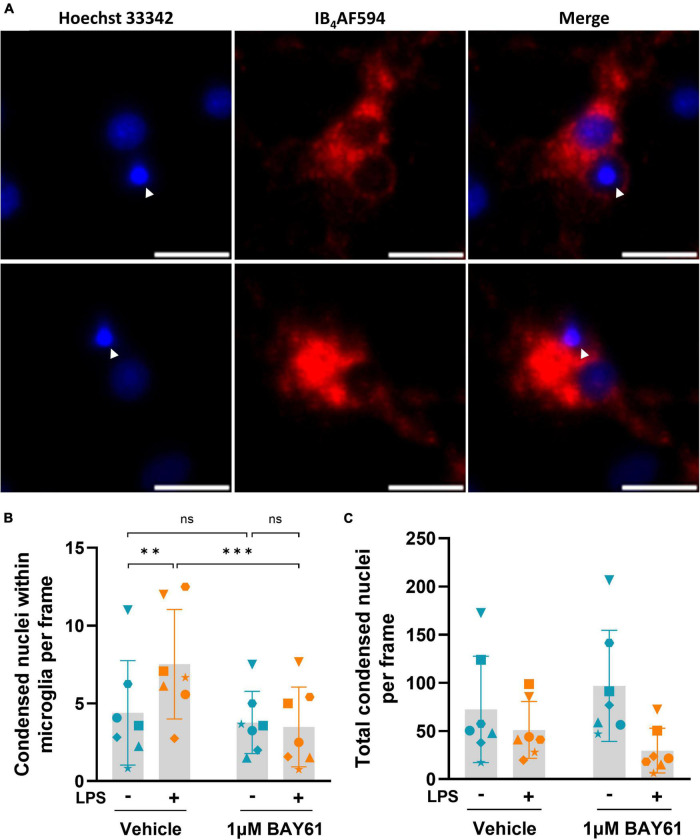
Syk inhibition prevents LPS-induced phagocytosis of neurons by microglia. **(A)** Representative fields of view depicting condensed nuclei (Hoechst 33342) within microglial membrane staining (IB_4_-AF594) from DIV7 images of neuron-glia cultures treated with 100 ng/ml LPS for 3 days. Arrowheads indicate condensed nuclei within microglia. Scale bars = 10μm. **(B)** Average counts of condensed nuclei within microglia per image in neuron-glia cultures treated ± BAY61 (1 μM) and ± LPS (100 ng/ml) for 3 days (DIV10). RM 2-way ANOVA with Šídák’s *post-hoc* test. **(C)** Average counts of total condensed nuclei per image in neuron-glia cultures treated ± BAY61 (1 μM) and ± LPS (100 ng/ml) for 3 days (DIV10). All panels: each datapoint represents the mean of three technical replicates. Number of biological repeats = number of datapoints per column. **p < 0.01, ***p < 0.001.

As Syk inhibition reduced microglial phagocytosis, and the LPS-induced neuronal loss in the neuronal-glial cultures is known to be mediated by microglial phagocytosis (Fricker et al., 2012a), it is possible that Syk inhibition prevented the LPS-induced neuronal loss by inhibiting microglial phagocytosis of stressed neurons. To test this, we reanalysed the images of LPS-induced neuronal loss ± BAY61 in the neuronal-glial cultures, quantified in Figure 1. As a proxy for neuronal soma phagocytosed by microglia, we examined condensed nuclei surrounded by microglia (Figure 7A), although at this resolution we cannot know that all these events are due to phagocytosis. Most of these condensed nuclei will be stressed neurons, as we have previously shown that the nuclei of live-but-stressed neurons condense (Hornik et al., 2016) and that the nuclei of phagocytosed neurons condense (Neher et al., 2014). With these caveats, we found that LPS treatment caused a significant increase in the number of condensed nuclei within microglia, and this was prevented by BAY61 treatment (Figure 7B). Importantly, this effect was not correlated with changes in the total number of condensed nuclei in the images, which could have otherwise explained changes in the number of condensed nuclei within microglia (Figure 7C). Overall, this suggests that Syk inhibition prevents LPS-induced phagocytosis of neurons.

## Discussion

In this study, Syk activity is shown to mediate inflammatory neuronal loss. Two structurally unrelated Syk inhibitors completely prevented LPS-induced neuronal loss in primary rat neuron-glia cultures, which we show is a microglia-dependent process. Syk inhibition caused some selective death of microglia in the absence of LPS. Given that Syk mediates signalling downstream of CSF1R (Bohlen et al., 2017), and that CSF1R inhibition or loss-of-function selectively depletes microglia (Erblich et al., 2011; Elmore et al., 2014), it is not surprising that Syk inhibition also depletes microglia. However, in the presence of LPS, Syk inhibition caused only mild and variable microglial depletion (0–30%); indeed, in the conditions of Figure 2, Syk inhibition caused no microglial depletion but fully prevented LPS-induced neuronal loss. Therefore, microglial depletion cannot explain the neuroprotection by Syk inhibitors in the presence of LPS.

Spleen tyrosine kinase inhibition also had no effect on the morphological transition induced by LPS, suggesting that Syk inhibition did not directly block inflammatory signalling. However, care should be taken when comparing this *in vitro* morphological data to *in vivo* data, where resting microglia are ramified and become more amoeboid with activation (e.g., Ennerfelt et al., 2022). In our *in vitro* model and as seen by others (Balion et al., 2022), resting microglia adopt a largely unramified morphology and become more adherent upon inflammatory stimulation, which in 2D cultures may resemble ramification, but may simply reflect greater adhesion to the coated plate (Wollmer et al., 2001). Irrespective of this difference of morphology *in vitro* and *in vivo*, Syk inhibition did not inhibit the morphological transition induce by LPS, and thus Syk inhibition does not block part of the inflammatory response of microglia to LPS.

Spleen tyrosine kinase inhibition did reduce LPS-induced IL-6 release (by about 45%) and mildly increased TNFα release (by about 80%). However, as the density of microglia was also reduced by Syk inhibition in these experiments (by about 30%), the amount of IL-6 released per cell was only mildly decreased, and the amount of TNFα released per cell was increased. Thus, Syk inhibition did not prevent inflammatory activation of microglia by LPS in conditions where it fully prevented LPS-induced neuronal loss. However, Syk inhibition did reduce total IL-6 levels (by about 45%, partly due to microglial depletion), and as IL-6 can be toxic to neurons (Conroy et al., 2004), this reduction might in principle contribute to the neuroprotection. In contrast, Kim et al. (2022) found that 100 nM BAY61 inhibited LPS-induced expression of TNFα and IL-1β in cultured BV2 microglia, and *in vivo* in mice BAY61 reduced LPS-induced TNFα and IL-1β expression. Thus, Syk inhibitors may be neuroprotective in part by reducing neuroinflammation, but this effect was limited in our culture system.

Spleen tyrosine kinase inhibition did reduce microglial phagocytosis in the presence and absence of LPS. BAY61 reduced phagocytosis of isolated synapses by microglia and reduced the synaptic loss (as measured by Homer1 protein levels) induced by low doses of LPS in neuron-glia cultures. This suggests that Syk inhibition reduces microglial phagocytosis of synapses. Synaptic loss/pruning is known to occur by microglial phagocytosis through complement receptor 3 (CR3; Hong et al., 2016) or TREM2 (Filipello et al., 2018; Linnartz-Gerlach et al., 2019), which induce phagocytosis through Syk, so the reduction in phagocytosis of synaptic material by Syk inhibition may be mediated by these or other Syk-dependent phagocytic receptors. The Syk inhibitor BAY61 has previously been shown to prevent synaptic loss in a tauopathy mouse model (Schweig et al., 2019), indicating that Syk inhibitors can prevent synaptic loss *in vivo* as well as in culture.

Previous studies have shown that microglial phagocytosis can mediate LPS-induced loss of entire neurons, and that inhibiting this phagocytosis in a variety of ways can prevent the neuronal loss (Fricker et al., 2012a,b; Neher et al., 2014). As Syk inhibition blocks phagocytosis induced by CR3, TREM2, Dectin-1, and Fcγ receptors (Crowley et al., 1997; Song et al., 2004; Gevrey et al., 2005; Mócsai et al., 2010; Scheib et al., 2012; Murakami et al., 2014; Yao et al., 2019; McQuade et al., 2020; Walbaum et al., 2021), this inhibition of microglial phagocytosis by Syk inhibitors appears sufficient to explain the prevention of LPS-induced neuronal loss, although the reduced microglial density and IL-6 release may also contribute to the neuroprotection by Syk inhibitors. Consistent with Syk inhibition preventing neuronal loss by blocking microglial phagocytosis of neurons, we found that LPS increased the number of condensed nuclei found within microglia, and Syk inhibition prevented this. We have previously shown that the neurons can undergo reversible nuclear condensation prior to phagocytosis (Hornik et al., 2016) and are condensed after phagocytosis by microglia (Neher et al., 2014). Note that the imaging is not sufficient to be sure that all the condensed nuclei belong to neurons and that all the condensed nuclei are phagocytosed by, rather than co-localised with, microglia. However, most such events are likely to be due to microglial phagocytosis of neurons.

Although we showed that LPS-induced neuronal loss requires microglia, we can’t rule out that the Syk inhibitors prevented neuronal loss by acting on Syk in neurons, rather than Syk in microglia. However, as the LPS-induced neuronal loss requires microglia, we cannot investigate any such potential protection in the absence of microglia. Syk inhibition also protected against spontaneous neuronal loss (in the absence of LPS) that occurred with age in the neuron-glia cultures. The mechanism of this loss and protection is unclear, but it does indicate that Syk inhibition is neuroprotective in conditions other than LPS-induced inflammation. Syk inhibition by BAY61 has previously been shown to increase baseline survival of hippocampal neurons in culture (Kim et al., 2022). Although LPS has widely been used to model inflammation, its use here to model acute neuroinflammation has limitations, as the neuroinflammation of neurodegenerative diseases is chronic and of complex origin. Care must therefore be exercised when extrapolating these results to humans disease, and future work will need to use other models and disease-relevant stimuli.

*In vivo*, Syk activity has been reported to be either beneficial or detrimental for neurons, depending on the model and disease parameters being studied. In a 5xFAD amyloid mouse model of AD, Syk promoted microglial phagocytosis and compaction of amyloid plaques, which reduced cognitive decline (Ennerfelt et al., 2022). However, Syk inhibition reduced neuronal and/or synaptic loss in mouse models of traumatic brain injury (He et al., 2022), stroke (Ye et al., 2020), LPS-induced inflammation (Kim et al., 2022), and tauopathy (Schweig et al., 2019). Notably, tauopathy more closely correlates with neuronal loss and cognitive decline in AD than amyloid pathology (King-Robson et al., 2021). It may be that Syk-mediated microglial phagocytosis is beneficial early on in AD by phagocytosis of amyloid, but detrimental later on by excessive phagocytosis of synapses and neurons. More generally, phagocytosis has important physiological and protective functions, but this may change depending on the phagocytic target, the specific disease in question, and the stage of disease. It will therefore be an ongoing challenge for the field to determine when phagocytosis might be targeted with therapeutic benefit for any specific neuropathology.

## Conclusion

In this study we show that Syk inhibition can prevent LPS-induced neuronal loss, which is dependent on microglia. Syk inhibitors selectively induce cell death of microglia in the absence of LPS, but Syk inhibition has relatively little effect on microglial survival or morphological activation, or in the presence of LPS. However, Syk inhibition reduces microglial phagocytosis of beads, synapses and neurons, which may explain how Syk inhibitors prevent neuronal loss in neuron-glia cultures, although reduced microglial density and IL-6 release may also contribute. As commercial Syk inhibitors are safe and effective in humans, our data support that they might be clinically useful to prevent neurodegeneration for certain disease conditions; however, this will require further investigation.

## Data availability statement

The raw data supporting the conclusions of this article will be made available by the authors, without undue reservation.

## Ethics statement

The animal study was reviewed and approved by the University of Cambridge Animal Welfare and Ethical Review Body (AWERB).

## Author contributions

TB designed and carried out the experiments, performed the data analysis, and wrote the manuscript. GB supervised and designed this research and wrote the manuscript. Both authors contributed to the article and approved the submitted version.
