# Supplementary material for: Syk inhibitors protect against microglia-mediated neuronal loss in culture
Source: Front Aging Neurosci. 2023 Mar 15;15:1120952. doi: 10.3389/fnagi.2023.1120952 (PMC10050448; doi:10.3389/fnagi.2023.1120952)
Supplement: Supplementary file 1 [file Data_Sheet_1.PDF]

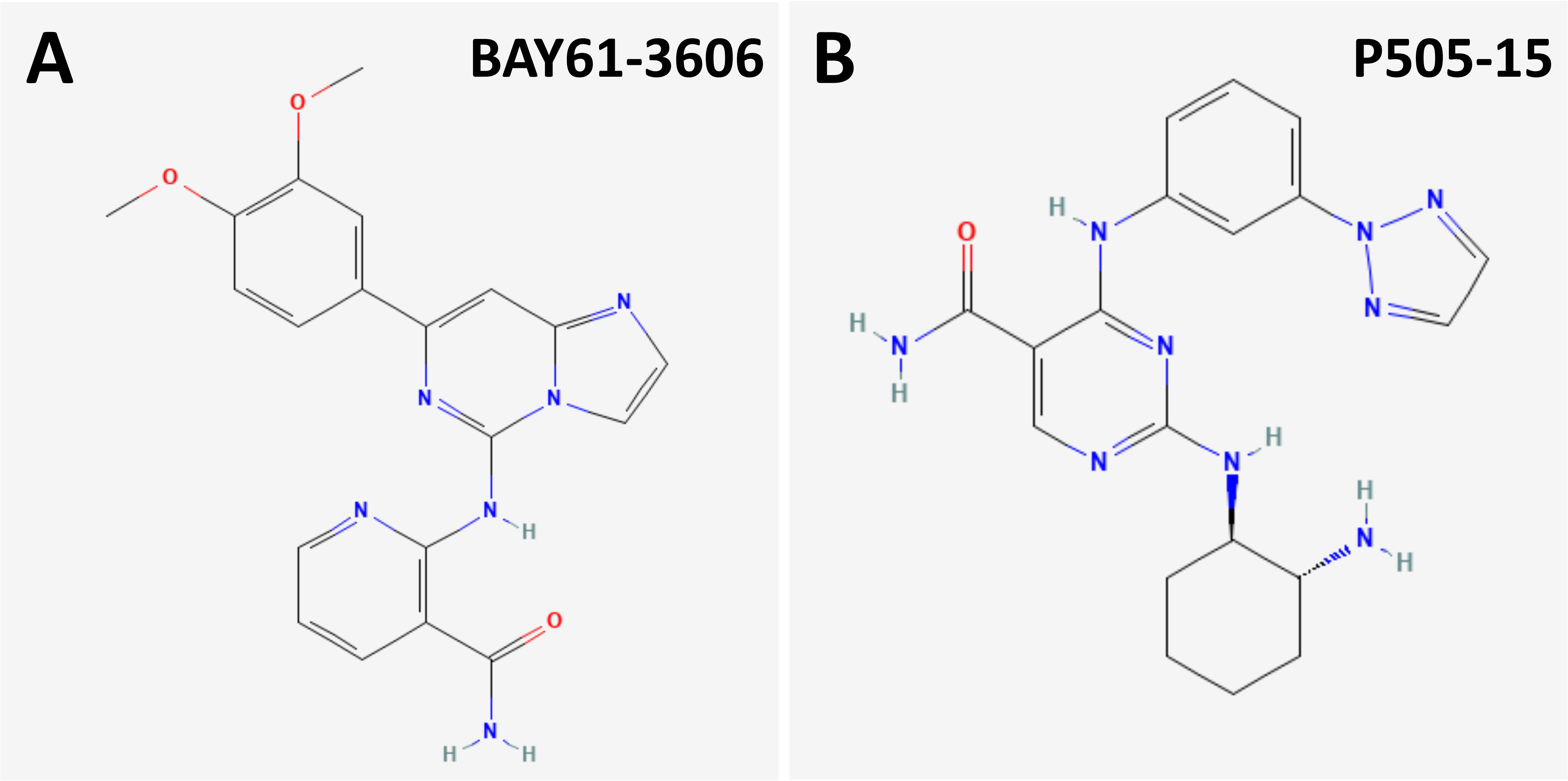

Figure S1. Syk inhibitors: BAY61-3606 and P505-15

A: Pubchem (Kim et al., 2021) structure for BAY61-3606 (BAY61). **B:** Pubchem structure for P505-15 (P505, also known as PRT062607). These inhibitors have distinct structural frameworks.

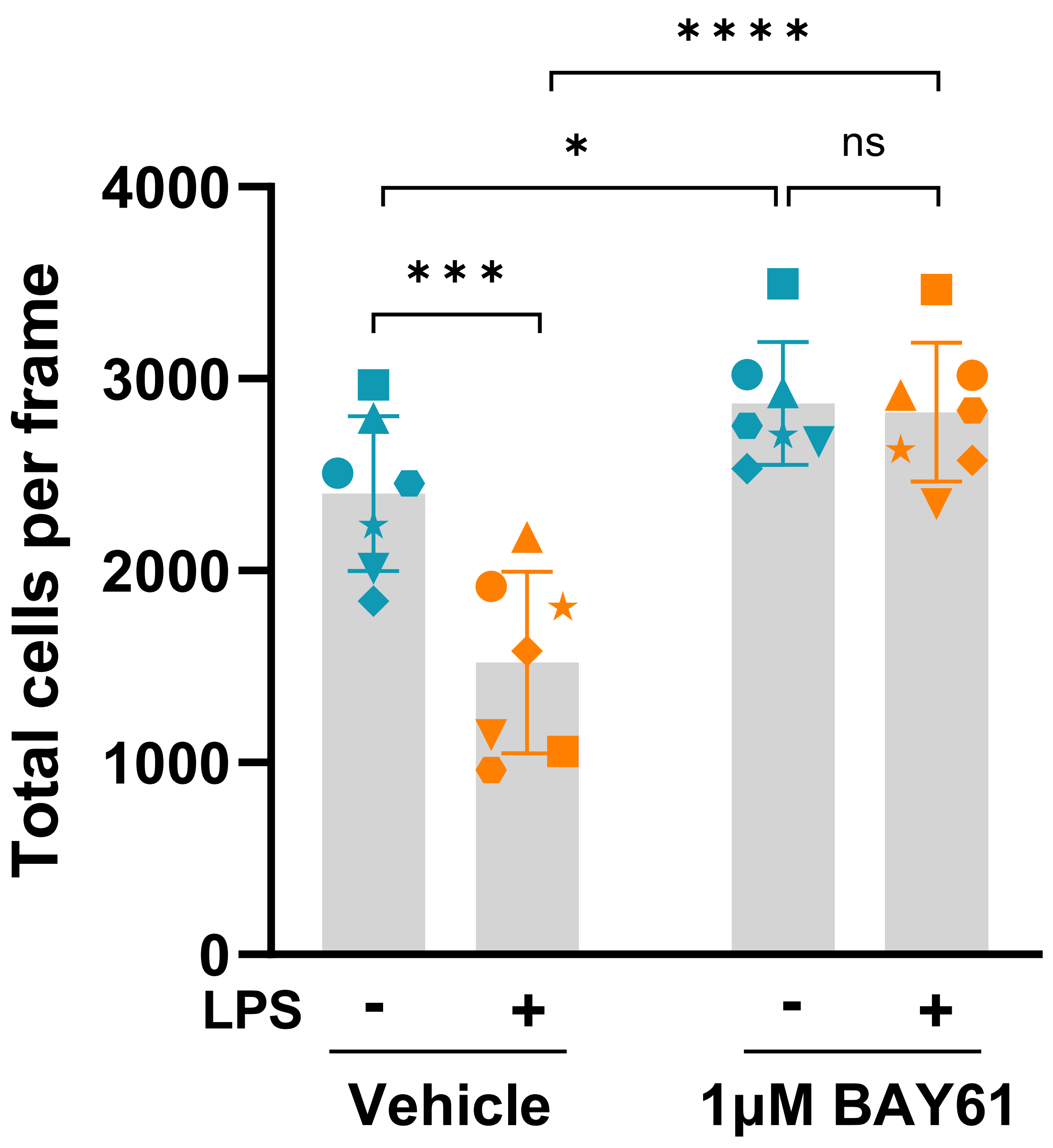

Figure S2. Total cell counts ± LPS and ± Syk inhibition in neuron-glia cultures

Average total cell counts per image in cultures treated ±BAY61 (1µM) and ±LPS (100ng/ml) for 3 days (DIV10). RM 2-way ANOVA with Šídák's post-hoc test.

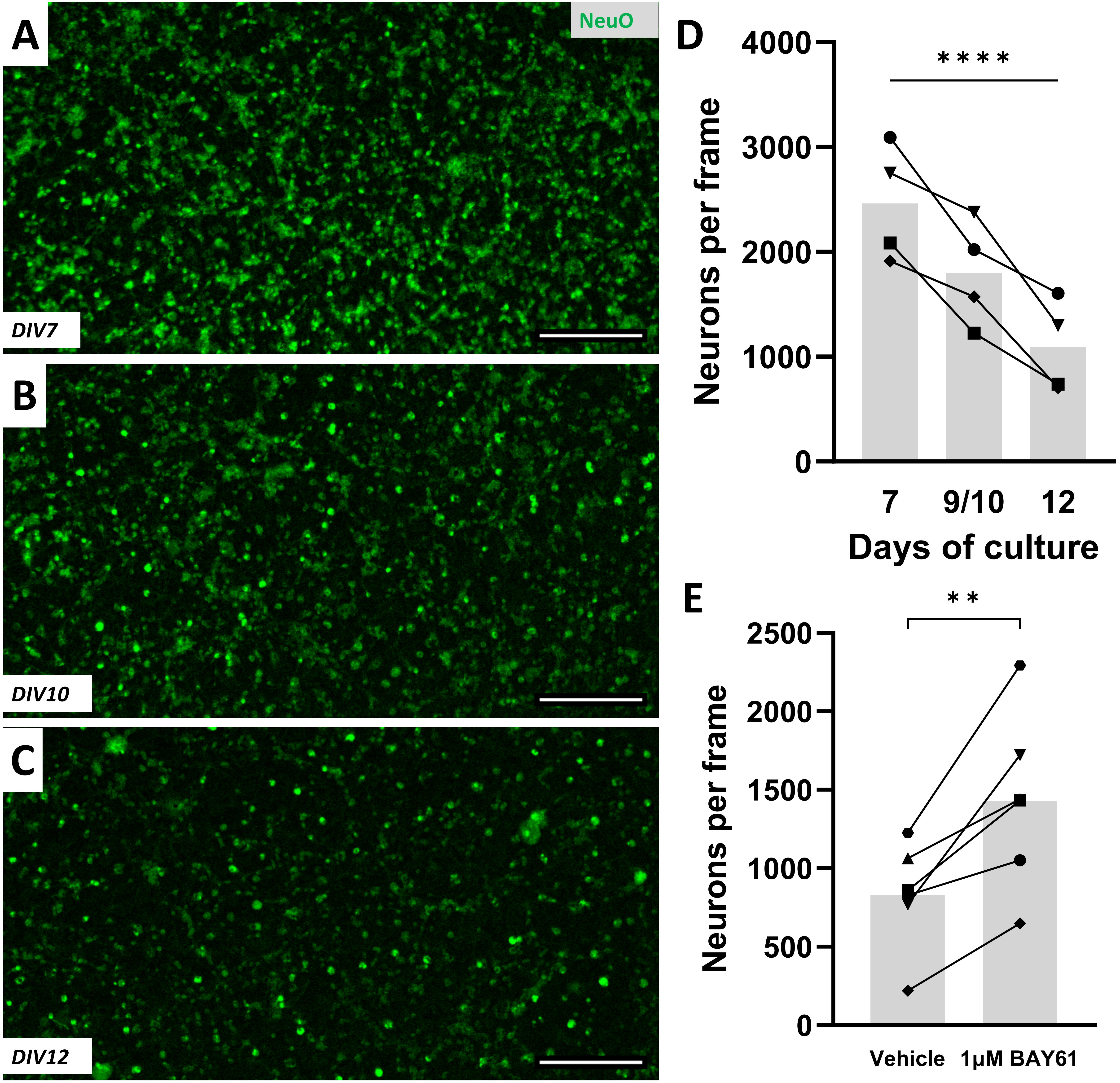

Figure S3. Syk inhibition reduces spontaneous neuronal loss. **A-C:** representative 10x images (cropped) of primary rat cerebellum neuron-glia cultures stained with NeuO (neurons) and imaged at *DIV7*, *DIV10*, and *DIV12*. Scale bars = 100µm. **D:** average *DIV7*, *DIV9-10*, and *DIV12* neuronal counts per image in untreated neuron-glia cultures. RM 1-way ANOVA with post-hoc test for linear trend. **E:** average *DIV12* neuronal counts per image in neuron-glia cultures treated ±BAY61 (1µM) for 3 days. Paired t-test. **All panels:** each datapoint represents the mean of 3 technical replicates. Number of biological repeats = number of datapoints per column. * $p<0.05$ ** $p<0.01$, **** $p<0.0001$.

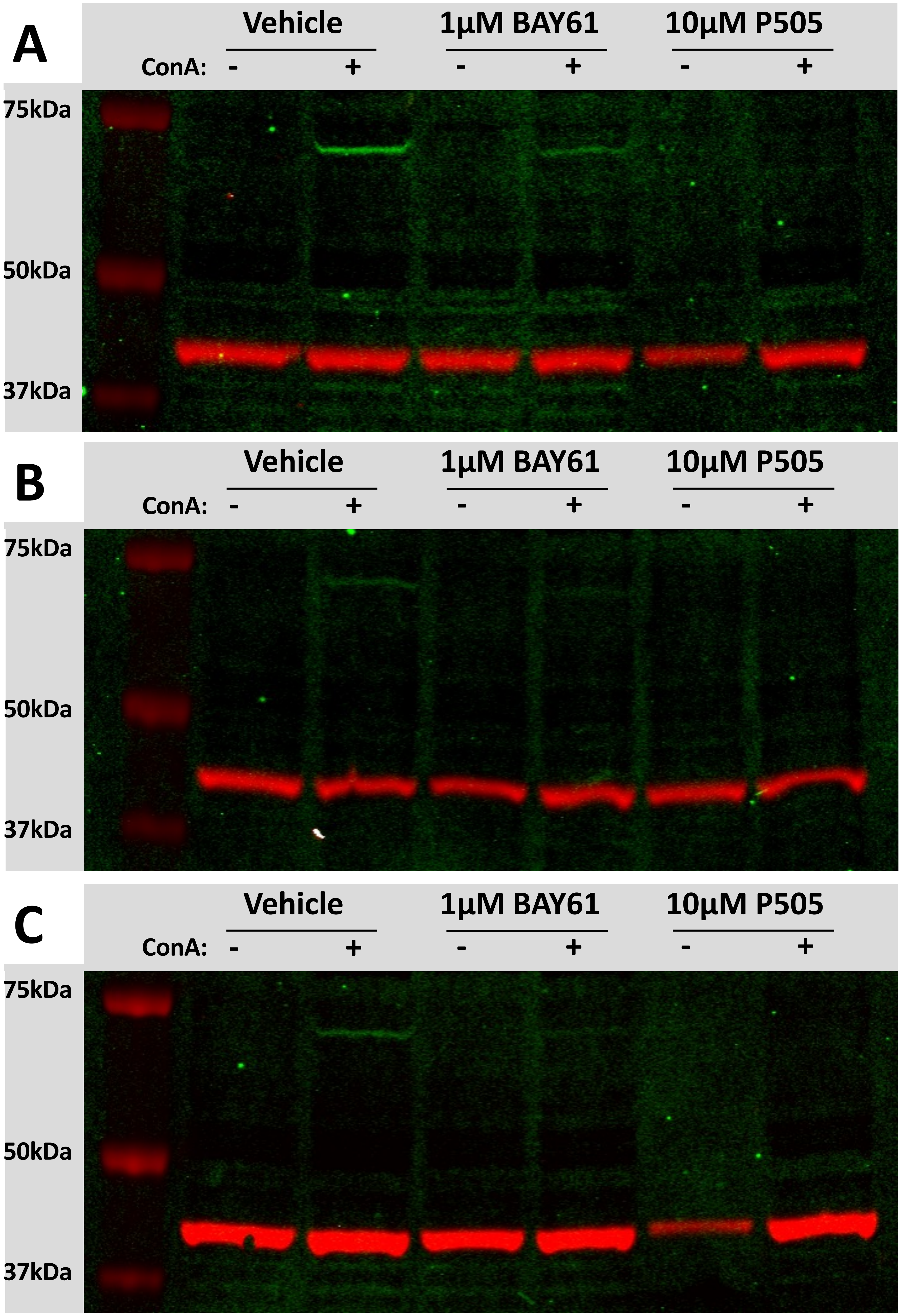

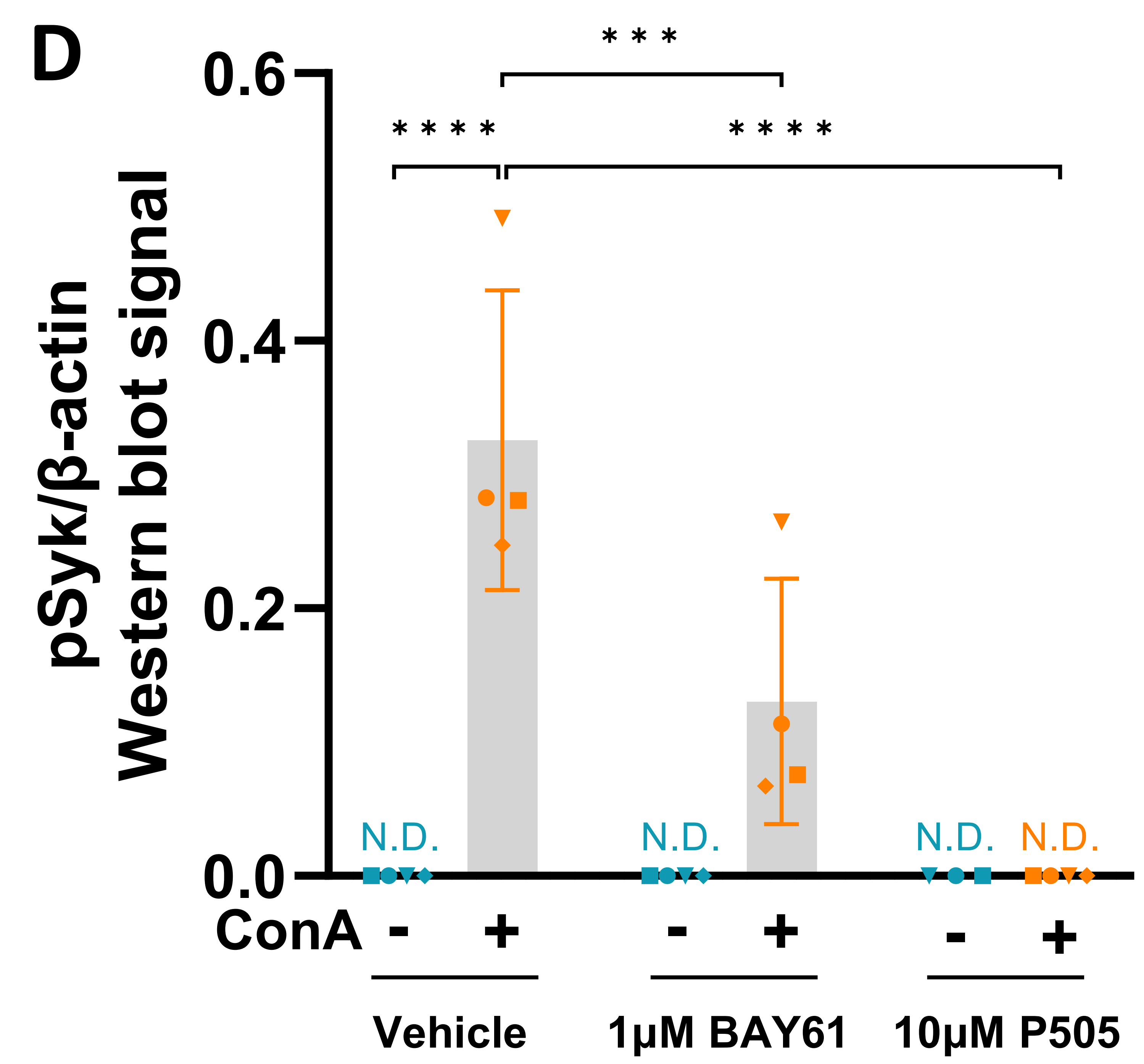

Figure S4. BAY61 and P505 inhibit Syk activity in mouse microglia

A-C: 3 biological repeats of Western blots on lysates from BV2 mouse microglia pre-treated ±BAY61 (1µM) or ±P505 (10µM) prior to 10 minutes of stimulation ±Concanavalin A (ConA; 50µg/ml). Red channel (700) = β-actin, green channel (800) = phospho-Syk (Tyr525/526). Actin and pSyk bands are as expected at 72kDa and 42kDa respectively. **D:** pSyk signal normalised against β-actin signal from the Western blots shown in **A-C** (and one further repeat, not shown). RM mixed-effects analysis with Šídák's post-hoc test. N.D. = not detectable.

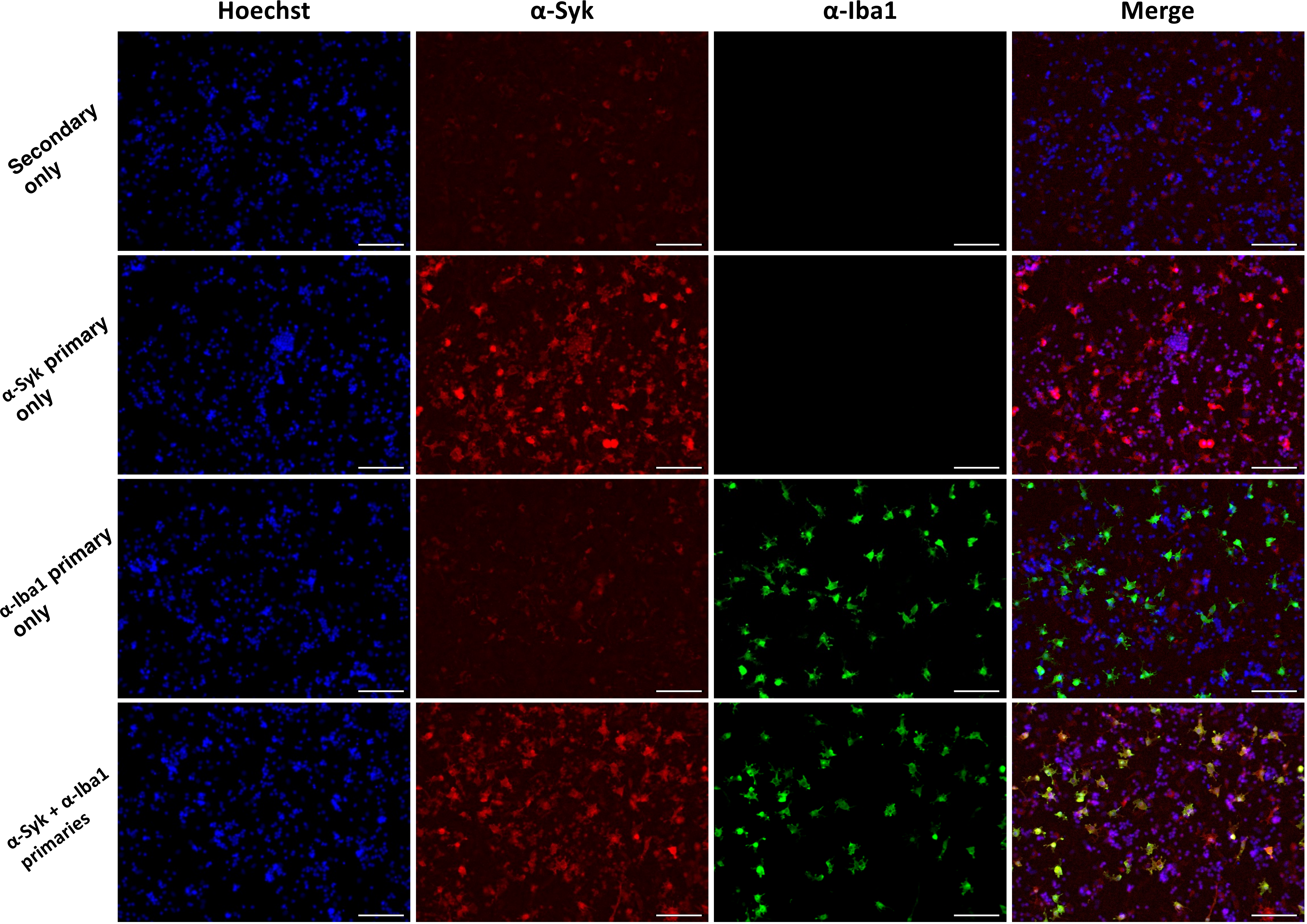

Figure S5. Syk is expressed by both neurons and microglia in rat cerebellum neuron-glia cultures. Representative 20x immunofluorescence images of primary rat neuron-glia cultures, fixed and stained with α-Syk (red) and α-Iba1 (green) antibodies, and Hoechst 33342 (DNA; blue). Scale bars = 100μm.

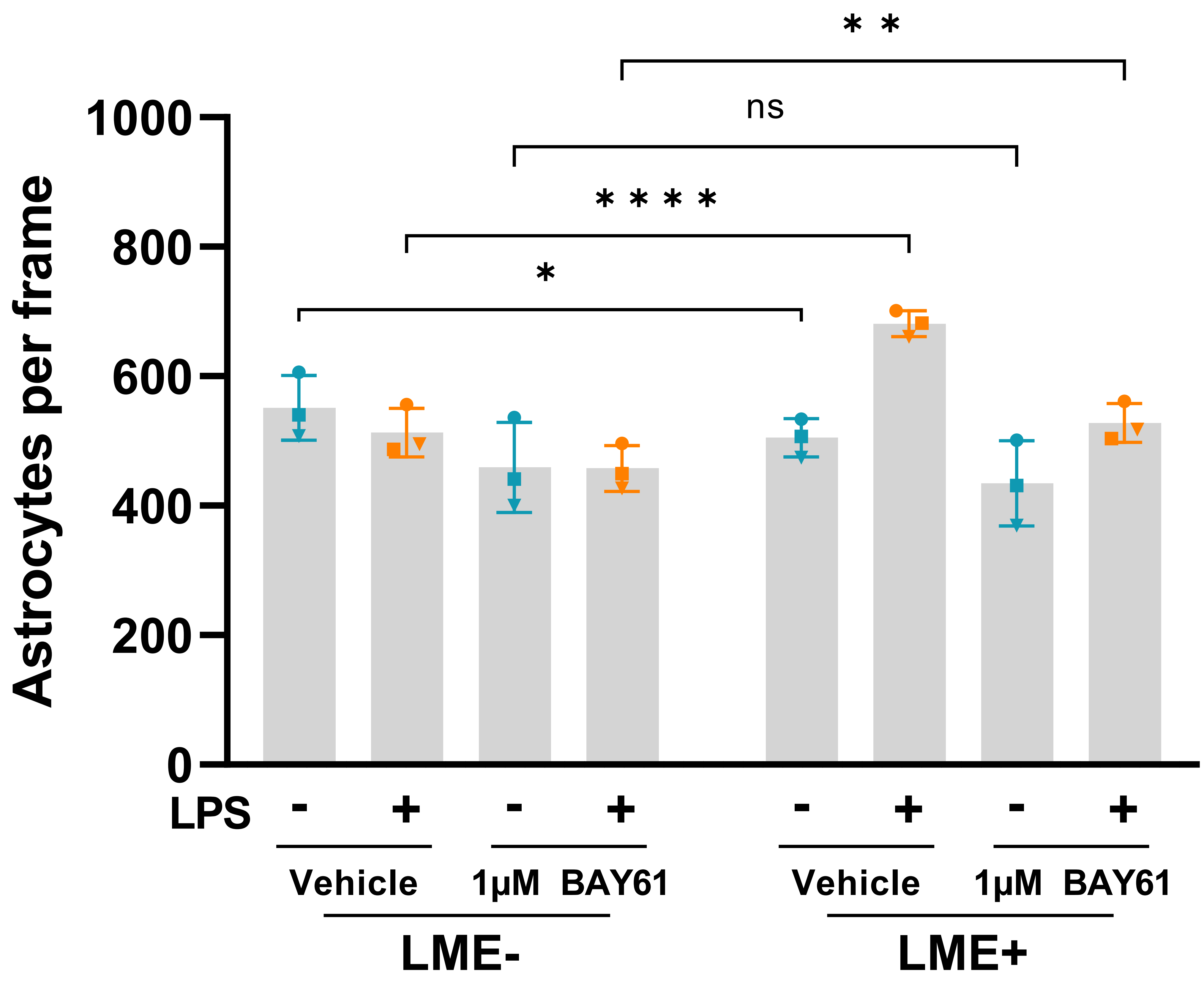

Figure S6. LME treatment does not strongly affect astrocyte populations.

Average *DIV10* astrocyte counts per image in neuron-glia cultures treated ±BAY61 (1μM) and ±LPS (100ng/ml) for 3 days, after previous ±LME treatment to deplete microglia. Each datapoint represents the mean of 3 technical replicates. Number of biological repeats = number of datapoints per column. * $p<0.05$ ** $p<0.01$, **** $p<0.0001$. RM 2-way ANOVA with Šídák's post-hoc test.

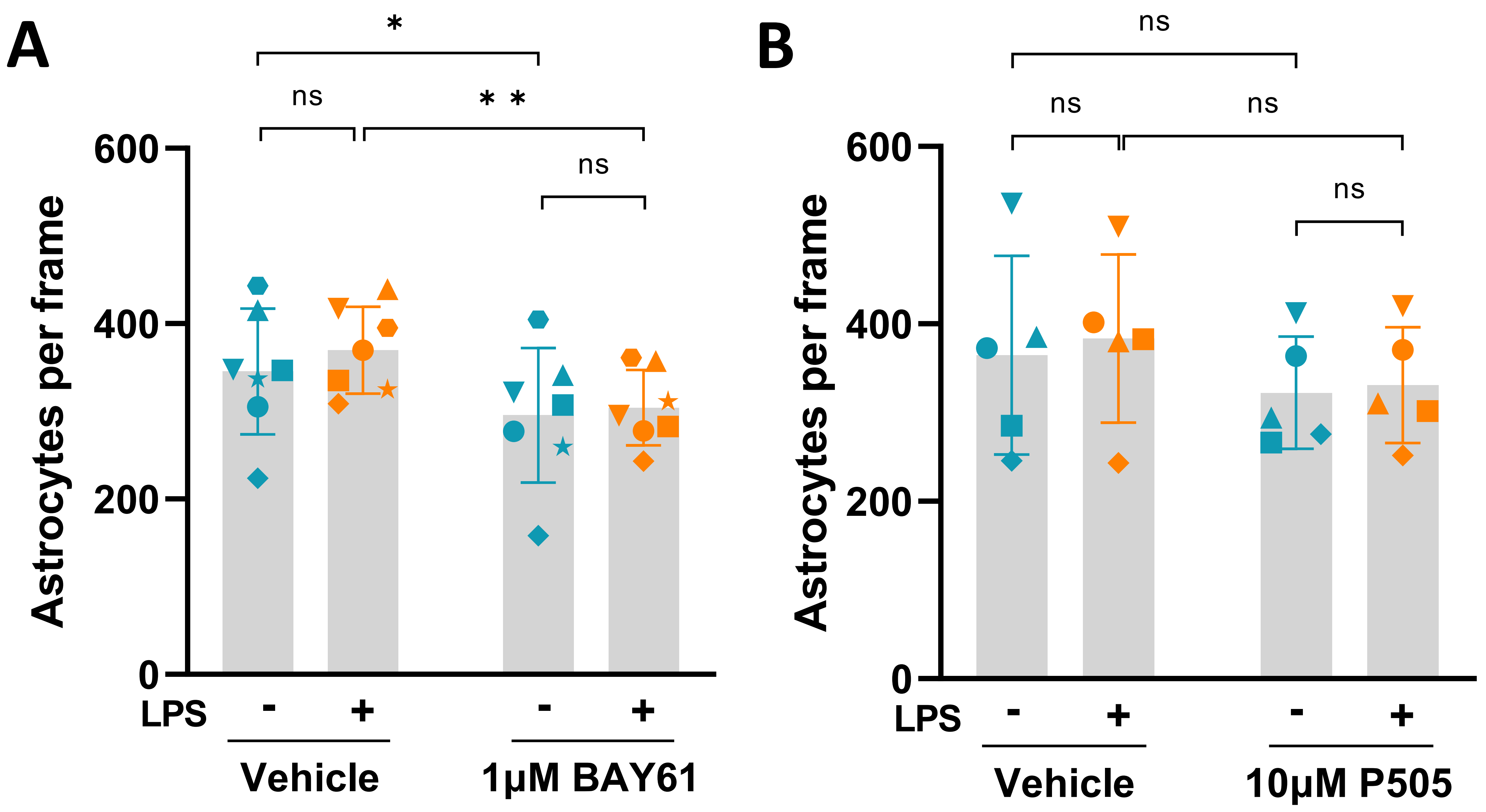

Figure S7. Syk inhibitors selectively deplete microglia relative to astrocytes

A: average *DIV10* astrocyte counts per image in neuron-glia cultures treated ±BAY61 (1μM) and ±LPS (100ng/ml) for 3 days. RM 2-way ANOVA with Šídák's post-hoc test. **B:** average *DIV10* astrocyte counts per image in neuron-glia cultures treated ±P505 (10μM) and ±LPS (100ng/ml) for 3 days. RM 2-way ANOVA with Šídák's post-hoc test. **All panels:** each datapoint represents the mean of 3 technical replicates. Number of biological repeats = number of datapoints per column. * $p<0.05$ ** $p<0.01$.

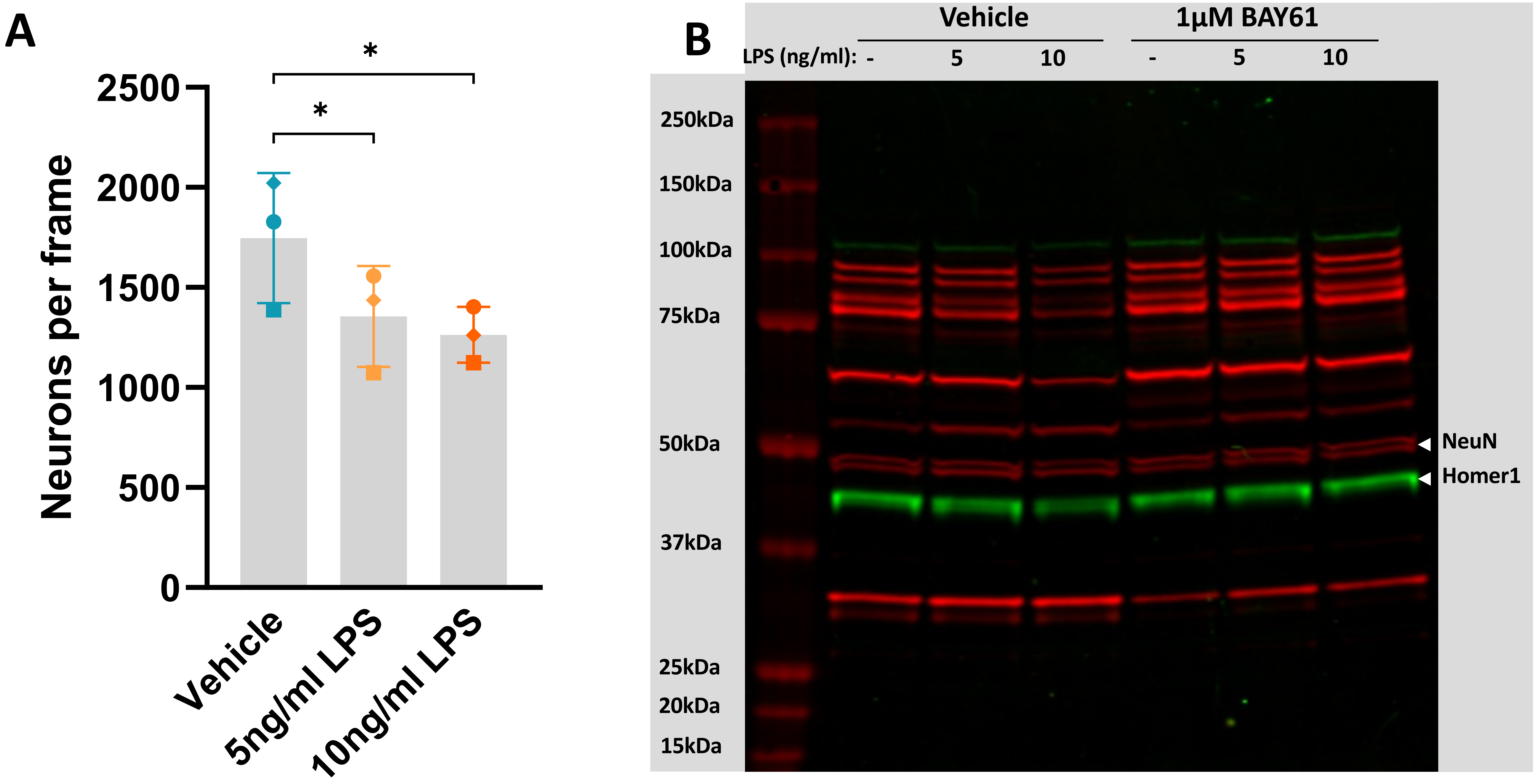

Figure S8: BAY61 protects against LPS-induced loss of Homer1 synaptic protein

A: average *DIV10* neuronal counts per image in cultures treated ±LPS (5-10ng/ml) for 3 days. RM 1-way ANOVA with Dunnett's post-hoc test. Each datapoint represents the mean of 3 technical replicates. 3 biological repeats. **B:** representative Western blot on lysates from *DIV10* neuron-glia cultures treated ±BAY61 (1µM) and ±LPS (5-10ng/ml) for 3 days. Red channel (700) = NeuN, green channel (800) = Homer1. Homer1 band is as expected at 40kDa, with a non-specific band observed >100kDa. NeuN bands are as expected at 46kDa and 48kDa, but various non-specific bands are also visible.
